# Rotational Thromboelastometry (ROTEM)-Guided Assessment of Hemorrhagic Disseminated Intravascular Coagulation in Septic Shock: A Case Report of Purpura Fulminans and Multiorgan Failure

**DOI:** 10.7759/cureus.115913

**Published:** 2026-09-07

**Authors:** Maria Dioselina Ruiz Barrera, Maria Alaciel Galvan Merlos, Cristina Elizabeth Madera Maldonado, Liliana Ponce Baños, Leticia Guerrero Navarrete, Fernando Villalobos Guerrero, Jorge Omar Castro Meza

**Affiliations:** 1 Adult Intensive Care Unit, Hospital Regional de Alta Especialidad de Ixtapaluca, Ixtapaluca, MEX; 2 Critical Care Medicine Department, Hospital Regional de Alta Especialidad de Ixtapaluca, Ixtapaluca, MEX

**Keywords:** disseminated intravascular coagulation (dic), fibrinogen replacement therapy, fulminans purpura, sepsis-associated disseminated intravascular coagulation (dic), viscoelastic tests

## Abstract

Sepsis-associated coagulopathy may progress rapidly from early thromboinflammation to overt disseminated intravascular coagulation (DIC) with mixed thrombotic and hemorrhagic manifestations. We report the case of a 30-year-old man with a history of methamphetamine use who presented after generalized tonic-clonic seizure activity, severe neurological impairment, hypoxemia, shock, and suspected sepsis. Initial evaluation showed severe metabolic derangement, acute kidney injury, aspiration risk during airway management, and suspected central nervous system infection. During intensive care unit evolution, the patient developed refractory septic shock, severe hypoxemic respiratory failure, rhabdomyolysis, recurrent hypoglycemia, profound mixed acidosis, anuric acute kidney injury requiring continuous renal replacement therapy, progressive thrombocytopenia, gastrointestinal bleeding, airway bleeding, catheter-site bleeding, and rapidly progressive violaceous bullous and necrotic skin lesions compatible with purpura fulminans. Clinical and laboratory findings were consistent with overt DIC with a prominent hemorrhagic phenotype. Serial rotational thromboelastometry (ROTEM) assessment demonstrated progressive coagulation failure, with late fibrin-based thromboelastometry (FIBTEM) findings showing severely reduced fibrin-based clot firmness consistent with fibrinogen-dependent clot failure. Hemostatic management included fibrinogen replacement, blood component therapy, tranexamic acid, and local hemostatic measures. Despite invasive mechanical ventilation, dual vasopressor therapy, corticosteroids, antimicrobial therapy, bicarbonate therapy, continuous renal replacement therapy, and targeted hemostatic resuscitation, the patient progressed to irreversible shock and cardiac arrest without return of spontaneous circulation. This case highlights the value of integrating clinical bleeding phenotype, conventional coagulation testing, DIC scoring, and viscoelastic assessment in critically ill septic patients with rapidly evolving coagulopathy.

## Introduction

Sepsis is a life-threatening syndrome caused by a dysregulated host response to infection and may progress to septic shock, multiorgan failure, and death. In critically ill patients, this process involves not only systemic inflammation but also endothelial injury, microcirculatory dysfunction, mitochondrial impairment, tissue hypoperfusion, metabolic derangement, and activation of coagulation pathways. Sepsis-associated coagulopathy represents a dynamic continuum that may begin as thromboinflammation or immunothrombosis, in which coagulation activation initially contributes to host defense, but may later become maladaptive and progress to overt disseminated intravascular coagulation (DIC). This transition is clinically important because septic DIC may present with predominantly thrombotic, hemorrhagic, or mixed phenotypes, depending on the balance between thrombin generation, fibrinolytic dysregulation, platelet consumption, coagulation factor depletion, and endothelial injury. Once refractory shock, severe acidosis, respiratory failure, acute kidney injury, and coagulopathy coexist, each organ dysfunction may amplify the others, creating a self-perpetuating cycle of hypoperfusion, bleeding, microvascular thrombosis, and progressive multiorgan failure [1,2].

Purpura fulminans is a severe cutaneous manifestation that may occur in sepsis-associated DIC and reflects extensive dermal microvascular injury. It is characterized by rapidly progressive purpuric lesions, dermal microvascular thrombosis, hemorrhagic skin infarction, tissue necrosis, and frequent progression to multiorgan failure. Although classically associated with meningococcemia, it may occur in other forms of severe bacterial sepsis and should be interpreted as a clinical marker of systemic endothelial and microvascular collapse rather than as an isolated dermatologic condition. Its pathophysiology reflects uncontrolled thrombin generation, endothelial activation, platelet and coagulation factor consumption, impaired fibrinolysis, and acquired dysfunction of endogenous anticoagulant pathways, particularly the protein C/protein S axis. This mechanism explains the paradoxical coexistence of cutaneous thrombosis and necrosis with active bleeding from mucosal surfaces, the gastrointestinal tract, the airway, puncture sites, or catheter insertion sites. Therefore, the presence of purpura fulminans in septic shock should alert clinicians to advanced thromboinflammatory failure and a high-risk mixed thrombotic-hemorrhagic phenotype [3,4].

Conventional coagulation tests, including platelet count, prothrombin time, activated partial thromboplastin time, fibrinogen concentration, and D-dimer, remain essential for diagnosing and monitoring DIC. However, these tests provide limited information regarding whole-blood clot initiation, clot propagation, fibrin polymerization, platelet-fibrin interaction, clot firmness, and fibrinolysis. Rotational thromboelastometry (ROTEM) provides a bedside assessment of whole-blood clot dynamics and may complement conventional coagulation testing when characterizing the hemostatic phenotype of actively bleeding critically ill patients. In particular, fibrin-based thromboelastometry (FIBTEM) evaluates the fibrinogen-dependent contribution to clot firmness by minimizing the platelet component of the clot, which may be especially useful when hypofibrinogenemia or functional fibrinogen failure contributes to hemorrhage. Serial ROTEM assessment may therefore complement conventional laboratory testing and support phenotype-directed hemostatic resuscitation in septic patients with rapidly evolving coagulopathy. We report the case of a 30-year-old man with refractory septic shock complicated by overt hemorrhagic DIC, clinically suspected purpura fulminans, multiorgan failure, and serial ROTEM evidence of evolving fibrinogen-dependent clot failure [5,6].

## Case presentation

Patient information and initial presentation

A 30-year-old man, with no previously documented chronic degenerative diseases, was brought to the emergency department by prehospital personnel after being found unconscious in a public area. According to collateral information obtained from relatives and witnesses, the patient had been working as a garbage collector and was reportedly found after an episode compatible with generalized tonic-clonic seizure activity of undetermined duration. The event was associated with altered level of consciousness, failure to recover baseline neurological status, and emesis of gastric contents.

His relevant past history included tobacco use and chronic methamphetamine use, although the exact timing of the last exposure was uncertain. There was no known history of epilepsy, chronic kidney disease, chronic liver disease, hematologic disease, anticoagulant use, or known drug allergies. Because the prehospital circumstances were unclear and the patient initially arrived unidentified, the first diagnostic impression remained broad, including acute symptomatic seizure, sympathomimetic toxidrome, central nervous system infection, aspiration-related pulmonary injury, septic shock, rhabdomyolysis, acute kidney injury, and early sepsis-associated coagulopathy.

On arrival, the patient was critically ill, with severe neurological impairment and cardiorespiratory instability. Initial vital signs showed the following: blood pressure 87/39 mmHg, heart rate 184 beats/min, respiratory rate 39 breaths/min, peripheral oxygen saturation 87%, and axillary temperature 36°C. Neurologically, he had a Glasgow Coma Scale score of 6 points, without adequate recovery after the convulsive episode [7]. He was diaphoretic, acutely ill-appearing, and unable to protect the airway.

Physical examination was limited by the severity of presentation. Pupils were initially described as small and reactive, without clear gaze deviation. Pulmonary examination revealed impaired ventilation, predominantly at the right lung base. The abdomen was soft, without signs of peritoneal irritation. Skin examination showed extensive erythematous, violaceous, and bullous lesions involving the dorsal, lumbar, and gluteal regions, initially interpreted as burn-like or pressure-related lesions of unclear mechanism. These lesions later became clinically relevant in the context of septic DIC and suspected purpura fulminans.

Emergency stabilization and airway management

Given the low Glasgow Coma Scale score, hypoxemia, recurrent emesis, and high risk of aspiration, advanced airway management was performed shortly after admission. During induction, the patient developed abundant emesis of gastrobiliary content, estimated at approximately 1000 mL, requiring immediate suctioning of the oral cavity and airway. Orotracheal intubation was achieved on the first attempt with an 8.5 mm internal diameter endotracheal tube, and the patient was connected to invasive mechanical ventilation.

Initial ventilatory support required high inspired oxygen concentrations and positive end-expiratory pressure. Sedoanalgesia was initiated with midazolam and fentanyl. Due to hemodynamic instability, norepinephrine was started to maintain perfusion pressure. The initial clinical picture suggested severe systemic inflammatory response with shock physiology, acute hypoxemic respiratory failure, altered mental status, and early renal dysfunction.

Initial diagnostic approach

The initial diagnostic workup was directed toward neurological, infectious, toxicologic, pulmonary, renal, and metabolic etiologies. Based on the clinical history, the non-contrast cranial computed tomography (CT) scan showed no acute intracranial hemorrhage, mass effect, midline displacement, hydrocephalus, or any structural lesions sufficient to explain the patient's profound neurological deterioration. Representative images from the cranial CT are shown in Figure 1A-1D.

**Figure 1:**
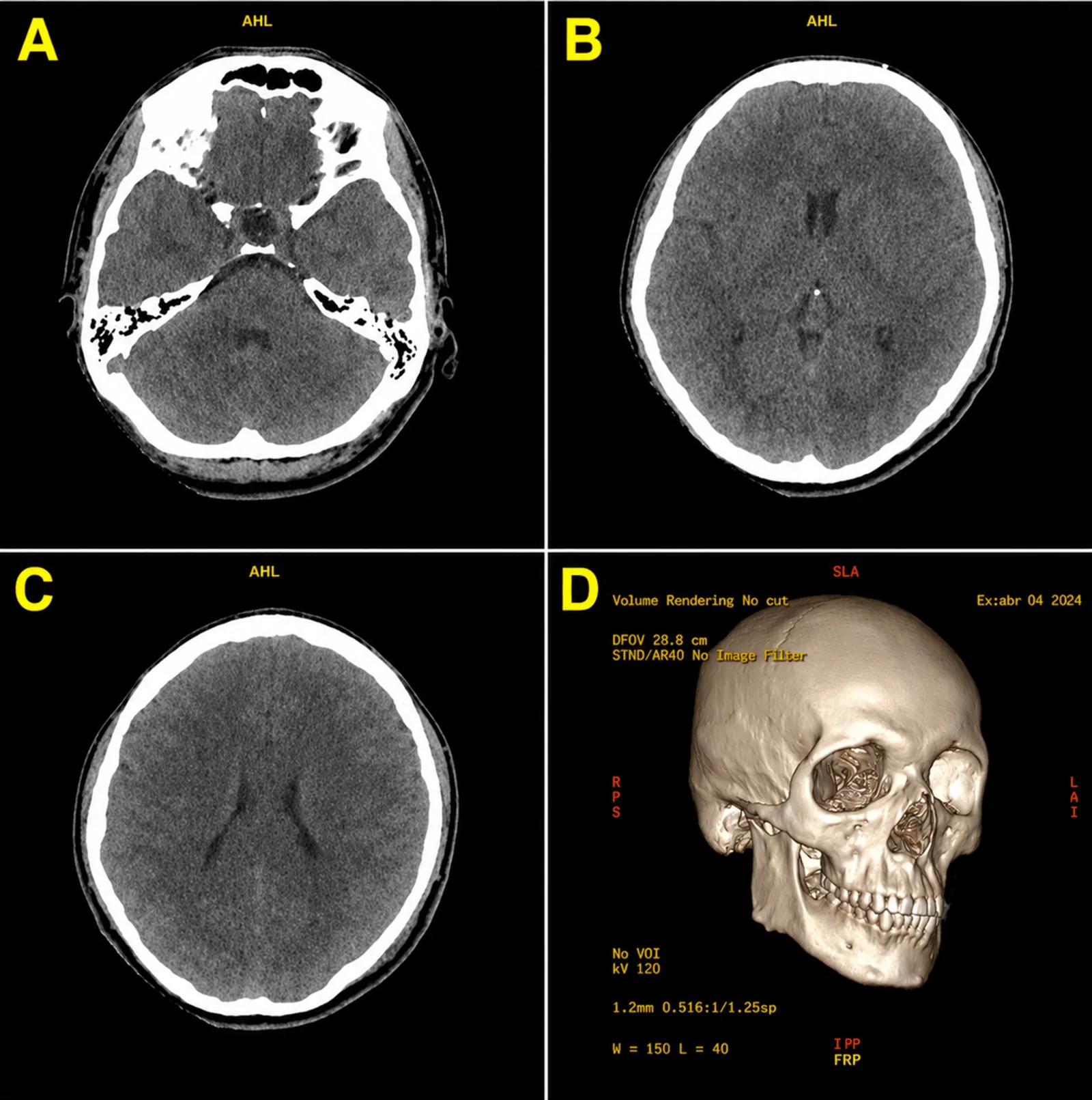
Representative CT images without contrast Panel A corresponds to the first representative image of the axial cranial CT. Panel B corresponds to the second representative image of the axial cranial CT. Panel C corresponds to the third representative image of the axial cranial CT. Panel D corresponds to the fourth representative image of the axial cranial CT. Overall, no acute intracranial hemorrhage, mass effect, midline displacement, hydrocephalus, or structural injury sufficient to explain the patient's neurological deterioration was identified. CT: computed tomography

Because of the combination of altered mental status, convulsive activity, leukocytosis, systemic inflammatory response, and absence of structural brain injury, lumbar puncture was performed to evaluate for suspected central nervous system infection.

The cerebrospinal fluid was macroscopically clear at the time of collection; however, preliminary microbiological reporting later described gram-positive cocci, supporting suspected neuroinfection. Final microbiological correlation was not fully available in the provided documentation, so the diagnosis was treated as probable central nervous system infection in the context of severe sepsis.

Toxicologic exposure remained part of the differential diagnosis because of the history of chronic methamphetamine use. This was clinically relevant in the context of generalized seizures, marked tachycardia, possible hypermetabolic state, rhabdomyolysis, acute kidney injury, and metabolic acidosis. However, the subsequent clinical evolution with refractory shock, progressive coagulopathy, multiorgan failure, and suspected purpura fulminans supported sepsis-associated thromboinflammatory collapse as the dominant process.

Pulmonary aspiration was also considered a major contributor to the clinical course. The patient had altered consciousness, seizure activity, and abundant emesis during airway management. According to the clinical record, chest imaging was reported as showing bilateral pulmonary infiltrates and right basal consolidation, supporting aspiration pneumonitis, aspiration pneumonia, or severe pulmonary infection as a possible septic focus.

The patient's initial clinical data and working diagnoses at admission are summarized in Table 1.

**Table 1:** Initial clinical data and working diagnoses Summary of the patient's baseline characteristics, prehospital presentation, initial vital signs, airway management, early diagnostic findings, and main working diagnoses at emergency department admission. The initial clinical scenario was broad and included suspected central nervous system infection, aspiration-related pulmonary infection, septic shock, sympathomimetic toxidrome, rhabdomyolysis, acute kidney injury, and early sepsis-associated coagulopathy. BP: blood pressure; HR: heart rate; RR: respiratory rate; SpO₂: peripheral oxygen saturation; T: temperature; CT: computed tomography; CSF: cerebrospinal fluid

Domain	Findings
Patient profile	Young adult male, no known chronic degenerative disease
Relevant exposure	Chronic methamphetamine use; timing of the last exposure uncertain
Prehospital event	Found unconscious after generalized tonic-clonic seizure activity
Initial neurological status	Glasgow Coma Scale score of 6 points
Initial vital signs	BP 87/39 mmHg, HR 184 bpm, RR 39 rpm, SpO₂ 87%, T 36°C
Airway	Orotracheal intubation due to neurological failure, hypoxemia, and aspiration risk
Aspiration risk	Abundant gastrobiliary emesis during airway management
Initial imaging	Cranial CT without acute structural lesion
CSF	Macroscopically clear; preliminary report with gram-positive cocci
Initial diagnostic considerations	Suspected neuroinfection, aspiration-related pulmonary infection, septic shock, sympathomimetic toxidrome, rhabdomyolysis, acute kidney injury, sepsis-associated coagulopathy

Admission to the intensive care unit (ICU)

The patient was admitted to the adult ICU under invasive mechanical ventilation, sedoanalgesia, and vasopressor support. At ICU admission, he remained critically ill with severe systemic inflammatory response, acute respiratory failure, metabolic acidosis, early acute kidney injury, thrombocytopenia, and suspected severe infection.

Initial arterial blood gas analysis showed severe acid-base derangement and impaired oxygenation, with pH 7.11, PaCO₂ 58 mmHg, PaO₂ 67 mmHg, bicarbonate 18.4 mmol/L, base excess -11.1 mmol/L, lactate 11.3 mmol/L, and a PaO₂/FiO₂ ratio close to 112. These findings were consistent with severe mixed acid-base disorder, predominantly high anion gap metabolic acidosis with a respiratory component, associated with tissue hypoperfusion and respiratory failure.

Initial laboratory assessment showed leukocytosis, thrombocytopenia, renal dysfunction, hyperuricemia, hypophosphatemia, hypomagnesemia, and elevated inflammatory biomarkers. Serum creatinine was elevated at 2.29 mg/dL. Platelet count was initially 105×10³/µL, with subsequent progressive decline. Procalcitonin was elevated, supporting severe bacterial infection or systemic inflammatory response. Creatine kinase values later increased markedly, consistent with rhabdomyolysis.

The initial ICU diagnoses included septic shock of suspected pulmonary and/or central nervous system source, severe acute hypoxemic respiratory failure, acute symptomatic seizures, possible sympathomimetic toxidrome, mixed acid-base disorder, acute kidney injury, rhabdomyolysis, thrombocytopenia, and extensive dorsal-gluteal cutaneous lesions of unclear mechanism.

Dermatologic findings and progression of cutaneous lesions

Cutaneous lesions were present early in the course and evolved rapidly during the period of hemodynamic and coagulation deterioration. Initially, the lesions were described as erythematous and bullous, with areas resembling second-degree burns or pressure-related injury. However, their progression toward violaceous discoloration, hemorrhagic bullae, epidermal detachment, necrotic-appearing plaques, and extensive purpuric changes raised concern for purpura fulminans in the context of septic DIC.

The serial dermatologic evolution is shown in Figure 2A-2D. On day 1, Figure 2A demonstrates multifocal dorsal and gluteal erythematous-violaceous lesions with areas of epidermal detachment and hemorrhagic exposed dermis. On day 2, Figure 2B and Figure 2C show progression of the lesions with larger violaceous plaques, hemorrhagic bullae, necrotic-appearing areas, and extension toward the flank and gluteal region. On day 3, Figure 2D demonstrates further coalescence of purpuric and necrotic lesions, with extensive hemorrhagic skin involvement compatible with a severe microvascular thrombotic and hemorrhagic process.

**Figure 2:**
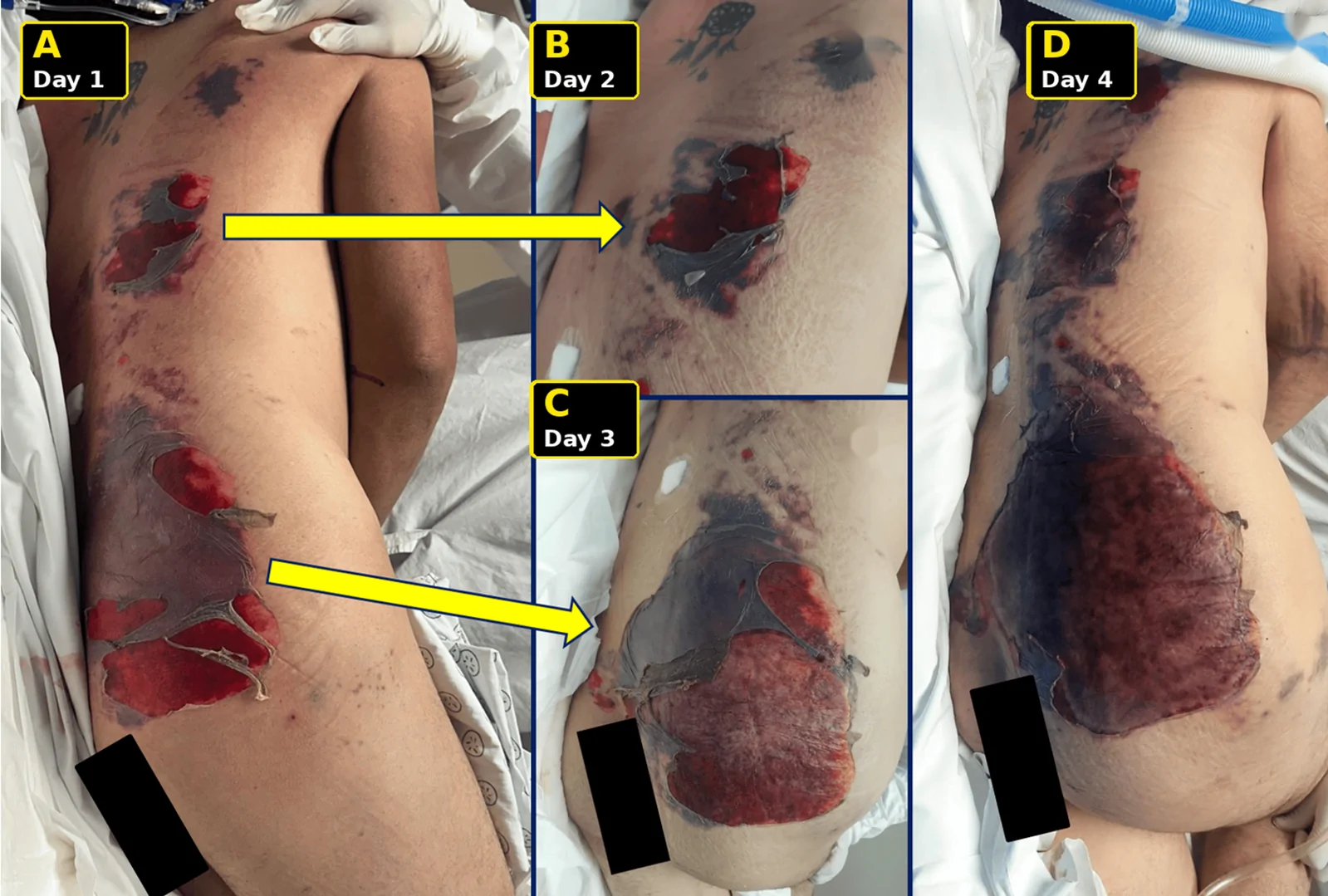
Serial progression of cutaneous lesions consistent with purpura fulminans associated with sepsis-induced disseminated intravascular coagulation Panel A corresponds to day 1 and shows dorsal multifocal lesions and erythematous-purplish gluteal lesions with epidermal detachment and exposed hemorrhagic dermis. Panel B corresponds to day 2 and shows progression towards larger purplish plaques, hemorrhagic bullae, necrotic-looking areas, and extension to the flank and gluteal region. Panel C corresponds to day 3 and shows greater enlargement of hemorrhagic bullae and necrotic-looking plaques with extensive purple involvement. Panel D corresponds to day 4 and shows coalescing purpuric and necrotic lesions with extensive hemorrhagic skin involvement, consistent with a severe dermal microvascular lesion in the context of septic disseminated intravascular coagulation. Yellow arrows indicate the temporal progression and enlargement of purpuric-necrotic lesions.

These findings were interpreted as clinically compatible with sepsis-associated purpura fulminans, particularly given the simultaneous development of refractory septic shock, overt DIC, active bleeding, thrombocytopenia, hypofibrinogenemia, and multiorgan failure. Although protein C and protein S levels were not documented in the available records, the clinical phenotype was consistent with acquired failure of endogenous anticoagulant pathways in severe sepsis.

Neurological evolution

From the neurological standpoint, the patient required deep sedation because of invasive mechanical ventilation, severe shock physiology, and profound metabolic instability. Sedation initially included midazolam and later required additional agents including propofol and dexmedetomidine according to the clinical course. Analgesia was maintained with fentanyl.

The neurological examination was difficult to interpret because of sedoanalgesia, severe metabolic acidosis, hypoxemia, uremia, possible toxicologic exposure, and refractory shock. Initially, the pupils were described as small and reactive. However, during clinical deterioration, the patient developed progressive pupillary abnormalities, including anisocoria. Later evaluations documented marked pupillary asymmetry and concern for severe neurological compromise in the setting of refractory shock, hypoxemia, profound acidosis, and multiorgan failure.

Bedside neuro-monitoring with optic nerve sheath assessment was performed during the clinical course. No immediate indication for hyperosmolar therapy was documented at that moment. The neurological prognosis remained poor because of the combined impact of suspected neuroinfection, postictal state, possible methamphetamine-associated toxicity, septic encephalopathy, hypoxemia, uremia, profound metabolic acidosis, and refractory shock.

Hemodynamic course and refractory septic shock

The patient evolved rapidly toward refractory septic shock. Although initial hemodynamic support was started with norepinephrine, vasopressor requirements increased progressively during ICU stay. Norepinephrine was titrated upward, and vasopressin was added due to persistent vasoplegia and hypoperfusion. Hydrocortisone was administered in the context of refractory septic shock.

Despite escalation of vasoactive support, the patient developed severe tissue hypoperfusion, evidenced by progressive mottling, prolonged capillary refill, rising lactate, severe metabolic acidosis, poor peripheral perfusion, and difficulty obtaining reliable pulse oximetry readings in the setting of distal hypoperfusion. Mottling score was reported as 5 points during severe deterioration, and capillary refill became markedly prolonged [8]. These findings suggested profound microcirculatory failure, not only macrocirculatory hypotension.

The shock was interpreted as predominantly septic/distributive, with possible contributing factors including central nervous system infection, aspiration-related pulmonary infection, severe systemic inflammation, endothelial injury, skin/soft tissue involvement, and metabolic consequences of seizure activity and methamphetamine exposure. As the disease progressed, hemorrhagic DIC, gastrointestinal bleeding, and severe coagulation failure likely further impaired oxygen delivery and worsened hemodynamic instability.

Respiratory course

The patient required invasive mechanical ventilation from the emergency department. The indication for intubation was multifactorial: severe neurological impairment, inability to protect the airway, hypoxemia, and high aspiration risk. The episode of abundant gastrobiliary emesis during induction increased concern for aspiration-related lung injury.

During ICU evolution, oxygenation worsened despite ventilatory support. According to the clinical record, chest imaging was described as showing bilateral infiltrates and right basal consolidation. These findings supported aspiration pneumonia, aspiration pneumonitis, or severe pulmonary infection as a probable contributor to sepsis and respiratory failure. The patient required high inspired oxygen fractions, ventilatory adjustments, and continued sedation to facilitate invasive ventilation.

Blood gas analyses demonstrated persistent severe acid-base abnormalities and hypoxemia. The respiratory course was therefore interpreted as severe acute hypoxemic respiratory failure in the context of septic shock, aspiration-related pulmonary injury, and metabolic instability. The coexistence of metabolic acidosis and respiratory acidosis complicated ventilation and contributed to global organ dysfunction.

Gastrointestinal and metabolic course

From the gastrointestinal standpoint, the patient initially remained fasting because of shock, airway instability, high vasopressor requirements, and risk of intolerance. A gastric tube was placed for decompression, and gastrobiliary output was documented. During ICU evolution, the patient developed gastrointestinal bleeding manifested by melena and dark gastrointestinal output. Episodes of melena were reported with variable volume, including clinically significant bleeding in the context of worsening coagulopathy.

The most likely mechanism was stress-related mucosal injury or erosive gastropathy associated with shock, mechanical ventilation, hypoperfusion, thrombocytopenia, and DIC. However, given the diffuse bleeding phenotype, gastrointestinal bleeding was considered part of systemic hemorrhagic coagulopathy rather than an isolated gastrointestinal event.

The metabolic course was severe. The patient developed recurrent hypoglycemia, including capillary glucose measurements reported as extremely low, requiring repeated dextrose administration and continuous glucose infusion. Hypoglycemia was interpreted as a marker of severe critical illness and possible impaired hepatic gluconeogenesis, high metabolic demand, systemic inflammation, and multiorgan failure.

Severe metabolic acidosis persisted despite resuscitative measures. Hyperlactatemia, renal failure, shock, rhabdomyolysis, and impaired tissue perfusion were considered major contributors. Sodium bicarbonate boluses and infusion were administered during episodes of profound acidemia. However, acidosis persisted and likely contributed to vasopressor resistance, impaired myocardial performance, worsening coagulopathy, and reduced response to hemostatic therapy.

Renal dysfunction, rhabdomyolysis, and continuous renal replacement therapy 

The patient developed severe acute kidney injury early during the hospital course. Initial creatinine was elevated, and urine output was markedly reduced. Despite fluid resuscitation and hemodynamic support, he progressed to anuric Kidney Disease: Improving Global Outcomes (KDIGO) stage 3 acute kidney injury [9].

The renal injury was considered multifactorial. Septic shock and hypoperfusion were major contributors, but rhabdomyolysis likely played an additional role. Creatine kinase values increased markedly during the ICU course, consistent with muscle injury. Potential contributors to rhabdomyolysis included generalized seizures, possible prolonged immobilization, methamphetamine exposure, hypoperfusion, and systemic inflammation. DIC-related renal microvascular thrombosis may also have contributed to renal dysfunction.

Given persistent anuria, severe metabolic acidosis, rising azotemia, and worsening clinical status, continuous renal replacement therapy was initiated in continuous veno-venous hemodiafiltration mode. However, continuous renal replacement therapy was challenging because of refractory shock and active bleeding. Circuit anticoagulation was limited or withheld because of severe thrombocytopenia and hemorrhagic DIC. Ultrafiltration was adjusted or suspended according to hemodynamic instability. Continuous renal replacement therapy was used as part of multiorgan support but could not reverse the underlying shock, endothelial failure, and consumptive coagulopathy.

Hematologic deterioration and overt DIC

The hematologic course became central to the case. The patient developed progressive thrombocytopenia, active bleeding, abnormal conventional coagulation tests, and ROTEM evidence of severe functional clot impairment. Initial platelet count was already reduced at approximately 105×10³/µL and subsequently declined. Conventional coagulation abnormalities included prolonged clotting times, markedly elevated fibrin-related markers, and hypofibrinogenemia according to the case presentation. The clinical phenotype included bleeding from multiple sites.

The patient developed upper gastrointestinal bleeding with melena, bleeding from the oral cavity, blood-tinged secretions from the endotracheal tube, oozing from venipuncture sites, and bleeding from central venous catheter and dialysis catheter insertion sites. This pattern was consistent with hemorrhagic DIC rather than isolated thrombocytopenia or isolated gastrointestinal bleeding.

The International Society on Thrombosis and Haemostasis (ISTH) DIC score was documented as compatible with overt DIC [10]. Some clinical documents reported an ISTH score of 5 points, while the case presentation described more severe coagulation abnormalities, including profound thrombocytopenia, markedly prolonged prothrombin time/activated partial thromboplastin time, extremely elevated D-dimer, fibrinogen below 100 mg/dL, and an ISTH DIC score of 8 points. Taken together, the patient's clinical and laboratory profile was consistent with advanced consumptive coagulopathy in the setting of severe sepsis and refractory shock.

The subsequent deterioration by organ system during the ICU course is summarized in Table 2.

**Table 2:** Major clinical deterioration by organ system Summary of the patient's progressive multiorgan deterioration during the intensive care unit course. The clinical evolution was characterized by severe neurological dysfunction, refractory septic shock, acute hypoxemic respiratory failure, anuric AKI requiring CRRT, severe metabolic failure, overt hemorrhagic DIC, and progressive cutaneous lesions clinically compatible with purpura fulminans. GCS: Glasgow Coma Scale; CNS: central nervous system; KDIGO: Kidney Disease: Improving Global Outcomes; AKI: acute kidney injury; CRRT: continuous renal replacement therapy; DIC: disseminated intravascular coagulation

Organ/system	Major findings	Clinical interpretation
Neurological	Generalized seizures, GCS 6, suspected neuroinfection, later anisocoria	Multifactorial neurological injury: suspected CNS infection, toxicologic exposure, hypoxemia, acidosis, septic encephalopathy
Hemodynamic	Refractory shock, escalating norepinephrine, vasopressin, mottling score 5, prolonged capillary refill	Refractory septic shock with severe microcirculatory failure
Respiratory	Invasive mechanical ventilation, aspiration risk, bilateral infiltrates, right basal consolidation, severe hypoxemia	Acute hypoxemic respiratory failure, probable aspiration-related pulmonary sepsis/injury
Renal	Anuria, creatinine elevation, KDIGO 3 AKI, CRRT	Severe sepsis-associated AKI, worsened by rhabdomyolysis and hypoperfusion
Metabolic	Hyperlactatemia, severe mixed acidosis, recurrent hypoglycemia	Severe shock-related metabolic failure
Hematologic	Thrombocytopenia, prolonged coagulation times, elevated D-dimer, hypofibrinogenemia, active bleeding	Overt hemorrhagic DIC
Dermatologic	Violaceous bullous lesions, hemorrhagic necrosis, progressive purpura	Suspected purpura fulminans associated with septic DIC

ROTEM assessment and serial viscoelastic evolution

Because of active bleeding, progressive thrombocytopenia, abnormal conventional coagulation tests, and suspected DIC, ROTEM was used to evaluate the functional coagulation phenotype. Serial ROTEM tracings demonstrated a dynamic evolution from severely abnormal clot formation to a late pattern dominated by marked hypocoagulability and fibrinogen-dependent clot failure.

The first available ROTEM tracing set, obtained on day 1, showed profoundly abnormal or absent clot formation in several assays, with extremely prolonged extrinsically activated thromboelastometry (EXTEM) and FIBTEM clotting times and very low amplitudes in intrinsically activated thromboelastometry (INTEM) and heparinase-modified thromboelastometry (HEPTEM), as shown in Figure 3A-3D. This suggested severe impairment of global clot formation, although preanalytical factors and sample quality should be considered when interpreting tracings with absent or nonmeasurable parameters.

**Figure 3:**
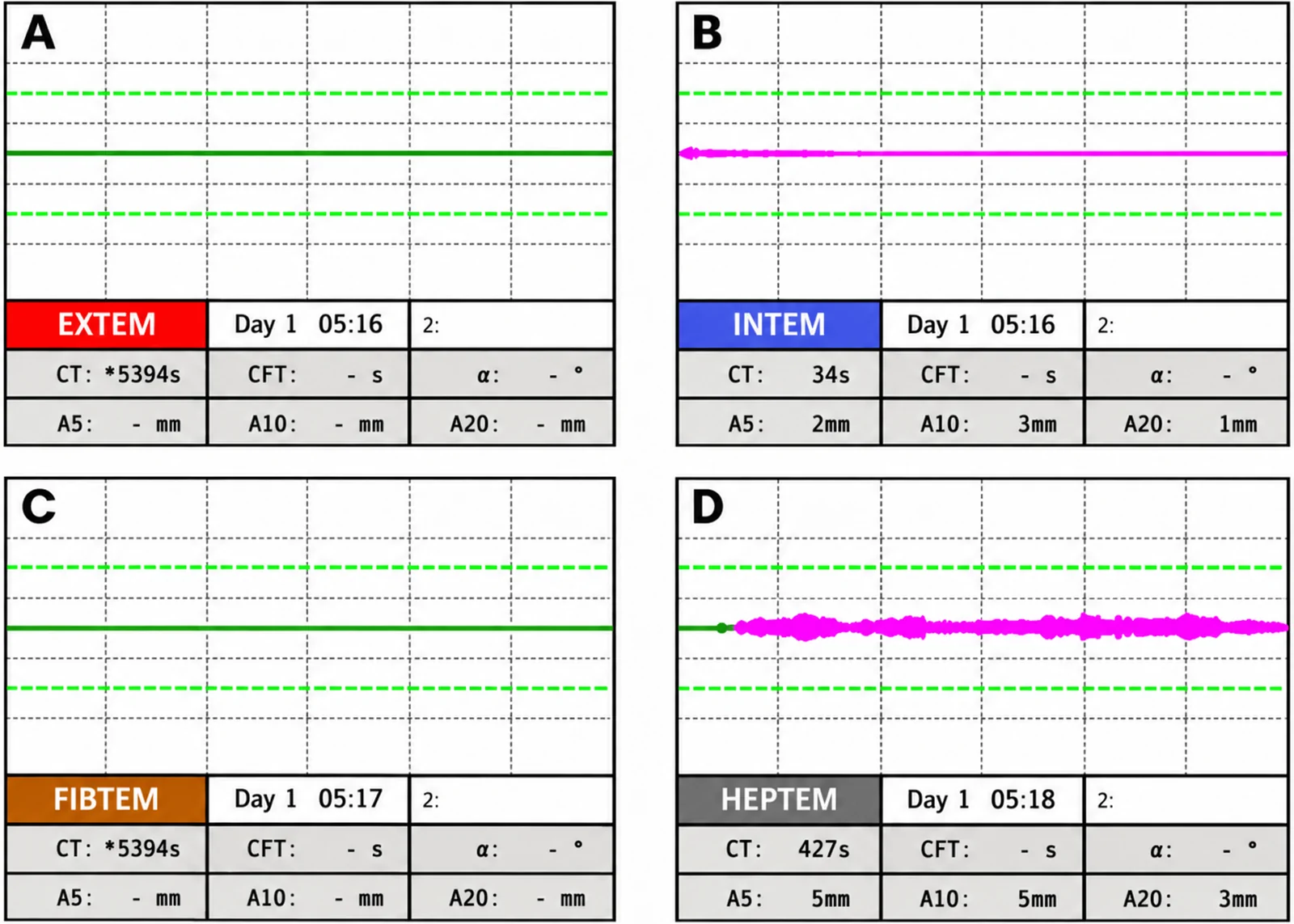
Early evaluation of ROTEM showing severely impaired clot formation during septic coagulopathy The ROTEM evaluation was obtained on day 1 between 05:16 a.m. and 05:18 a.m. Panel A shows EXTEM with CT >5394 s and nonmeasurable clot formation parameters, suggesting absence or severely impaired extrinsic formation. Panel B shows INTEM with CT 34 s and markedly reduced clot amplitudes, including A5 2 mm, A10 3 mm, and A20 1 mm, indicating a profound deterioration of clot firmness. Panel C shows FIBTEM with CT >5394 s and nonmeasurable clot amplitudes, indicating severely abnormally shaped fibrin-based clot formation. Panel D shows HEPTEM with CT 427 s and persistently low clot amplitudes, including A5 5 mm, A10 5 mm, and A20 3 mm, consistent with severe hypocoagulability. Overall, this early tracing demonstrated a profoundly abnormal viscoelastic profile consistent with severe coagulant failure in the context of septic shock, progression of disseminated intravascular coagulation, thrombocytopenia, acidosis, and active bleeding. Given the extreme values and nonmeasurable parameters, preanalytical factors or sample interferences should also be considered. ROTEM: rotational thromboelastometry; EXTEM: extrinsically activated thromboelastometry; INTEM: intrinsically activated thromboelastometry; FIBTEM: fibrin-based thromboelastometry; HEPTEM: heparinase-modified thromboelastometry; CT: clotting time; CFT: clot formation time; α: alpha angle; A5/A10/A20: clot amplitude at 5, 10, and 20 minutes

A second ROTEM tracing set obtained later on day 1 showed discordant findings. EXTEM clotting time was closer to normal, while INTEM clotting time was markedly prolonged and appeared to correct with HEPTEM, suggesting possible heparin effect, heparin contamination, or preanalytical interference. FIBTEM demonstrated preserved fibrin-based clot firmness at that time, with A5 15 mm, A10 17 mm, and A20 15 mm, as shown in Figure 4A-4D. This suggested that, during this intermediate phase, fibrinogen-dependent clot contribution was still relatively preserved despite systemic critical illness.

**Figure 4:**
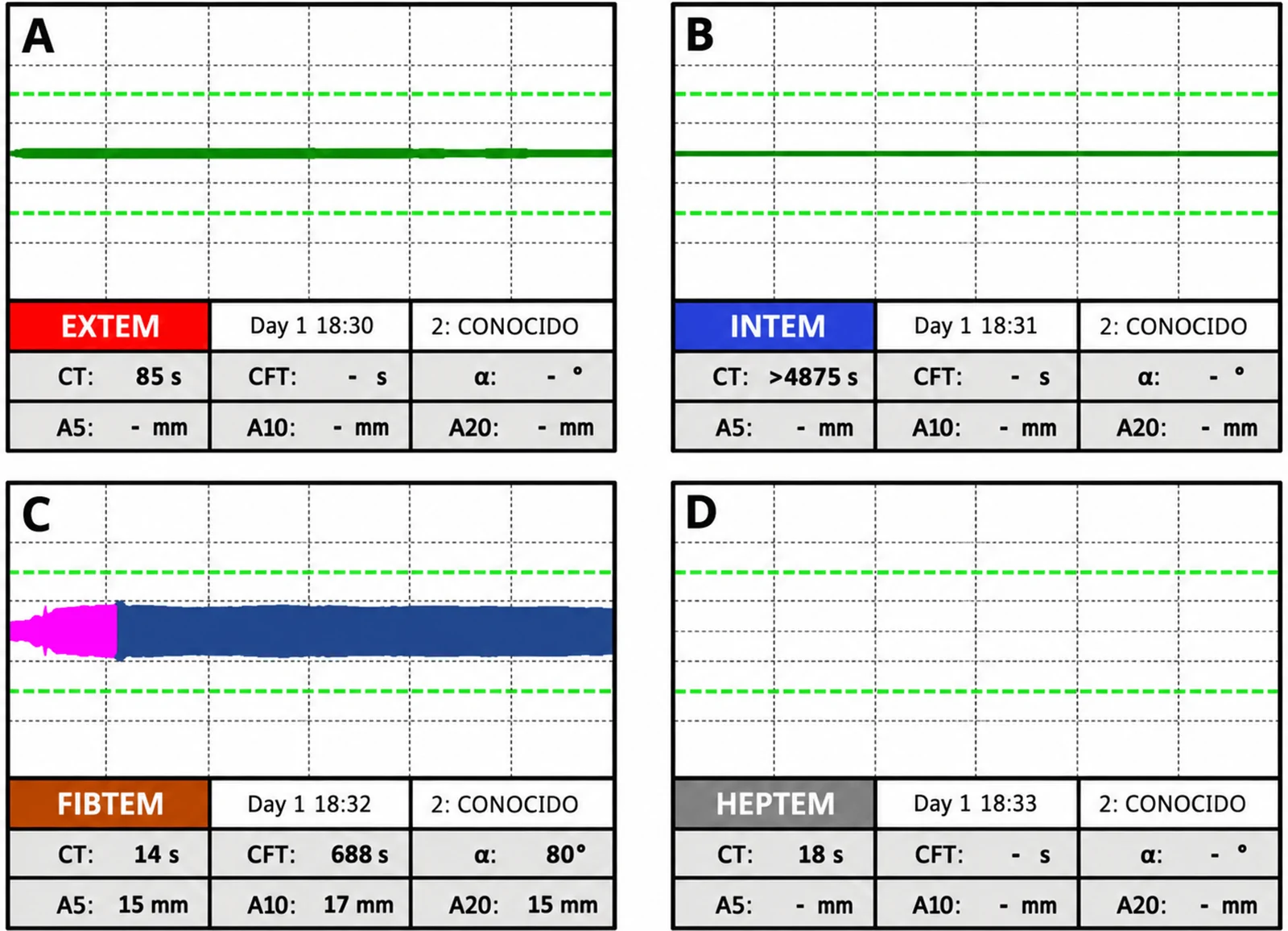
Intermediate ROTEM tracing with preserved FIBTEM clot firmness The ROTEM assessment was obtained on day 1 between 6:30 p.m. and 6:33 p.m. Panel A shows EXTEM with CT 85 s, suggesting a relatively preserved initiation of extrinsic clots. Panel B shows INTEM with markedly prolonged CT >4875 s, indicating a major intrinsic pathway delay. Panel C shows FIBTEM with CT 14 s, CFT 688 s, α angle 80°, and preserved clot amplitudes, including A5 15 mm, A10 17 mm, and A20 15 mm, indicating relatively preserved fibrinogen-dependent clot firmness at this time. Panel D shows HEPTEM with 18 s CT, suggesting an apparent correction of the INTEM abnormality and increasing the possibility of heparin effect, heparin contamination, or preanalytical interference. Overall, this intermediate tracing suggested a contribution of fibrin-based preserved clots despite discordant findings in intrinsic pathways. ROTEM: rotational thromboelastometry; EXTEM: extrinsically activated thromboelastometry; INTEM: intrinsically activated thromboelastometry; FIBTEM: fibrin-based thromboelastometry; HEPTEM: heparinase-modified thromboelastometry; CT: clotting time; CFT: clot formation time; α: alpha angle; A5/A10/A20: clot amplitude at 5, 10, and 20 minutes

By day 2, the ROTEM profile had deteriorated substantially and was consistent with advanced hypocoagulability. EXTEM showed prolonged clotting time and severely impaired clot propagation, with CT 326 s, CFT 795 s, alpha angle 24°, A5 11 mm, A10 17 mm, and A20 24 mm. INTEM also showed prolonged clotting time and delayed clot propagation, with CT 379 s, CFT 869 s, alpha angle 22°, A5 10 mm, A10 16 mm, and A20 23 mm. FIBTEM demonstrated markedly reduced fibrin-based clot firmness, with a CT of 295 s and amplitudes of 3 mm at A5, 4 mm at A10, and 4 mm at A20. This late pattern was consistent with markedly reduced fibrin-based clot firmness, suggesting impaired functional fibrinogen contribution, in the context of overt DIC and active bleeding, as shown in Figure 5A-5D.

**Figure 5:**
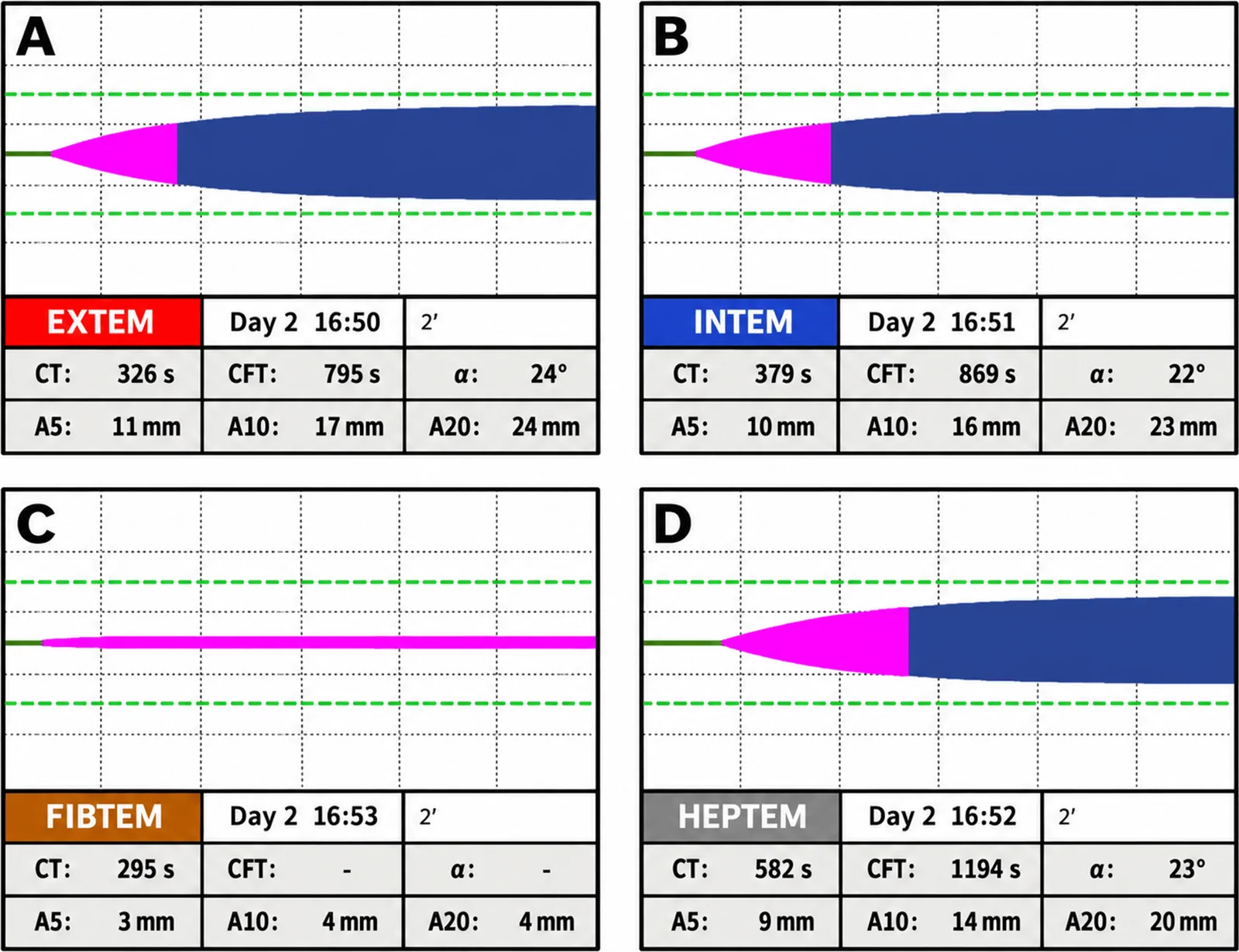
Late ROTEM tracing showing advanced hypocoagulability and fibrinogen clot-dependent failure The ROTEM assessment was obtained on day 2 between 4:50 p.m. and 4:53 p.m. Panel A shows EXTEM with CT 326 s, CFT 795 s, α angle 24°, and A5 11 mm, A10 17 mm, and A20 24 mm clot amplitudes, indicating delayed clot onset and impaired clot spread. Panel B shows INTEM with CT 379 s, CFT 869 s, α angle 22°, and clot amplitudes A5 10 mm, A10 16 mm, and A20 23 mm, consistent with global hypocoagulability. Panel C shows FIBTEM with a CT of 295 s and markedly reduced clot amplitudes (A5, 3 mm; A10, 4 mm; A20, 4 mm), consistent with severely reduced fibrin-based clot firmness and impaired functional fibrinogen contribution. Panel D shows HEPTEM with CT 582 s, CFT 1194 s, α angle 23°, and clot amplitudes A5 9 mm, A10 14 mm, and A20 20 mm, consistent with persistent coagulation impairment despite the heparinase channel. Overall, this late tracing demonstrated worsening global hypocoagulability with markedly reduced FIBTEM clot firmness, consistent with impaired functional fibrinogen contribution in overt disseminated intravascular coagulation with a hemorrhagic phenotype. ROTEM: rotational thromboelastometry; EXTEM: extrinsically activated thromboelastometry; INTEM: intrinsically activated thromboelastometry; FIBTEM: fibrin-based thromboelastometry; HEPTEM: heparinase-modified thromboelastometry; CT: clotting time; CFT: clot formation time; α: alpha angle; A5/A10/A20: clot amplitude at 5, 10, and 20 minutes

Table 3 shows the serial ROTEM findings and their clinical interpretation.

**Table 3:** Serial ROTEM findings and clinical interpretation Summary of serial rotational thromboelastometry findings obtained during the evolution of septic shock and overt DIC. Early tracings showed severely abnormal or absent clot formation with very low clot amplitudes. The evening tracing on day 1 demonstrated preserved FIBTEM clot firmness despite discordant INTEM/HEPTEM findings, suggesting possible heparin effect, heparin contamination, or preanalytical interference. By day 2, ROTEM showed global hypocoagulability with markedly reduced FIBTEM amplitudes, consistent with severely impaired fibrin-based clot firmness in overt DIC with active bleeding. ROTEM: rotational thromboelastometry; EXTEM: extrinsically activated thromboelastometry; INTEM: intrinsically activated thromboelastometry; FIBTEM: fibrin-based thromboelastometry; HEPTEM: heparinase-modified thromboelastometry; CT: clotting time; CFT: clot formation time; α: alpha angle; A5/A10/A20: clot amplitude at 5, 10, and 20 minutes; DIC: disseminated intravascular coagulation

Day/time point	Assay	Main values	Interpretation
Day 1, early ROTEM tracing	EXTEM	CT >5394 s; amplitudes not measurable	Severely abnormal/absent clot formation; interpret with clinical context and possible preanalytical factors
Day 1, early ROTEM tracing	FIBTEM	CT >5394 s; amplitudes not measurable	Severely abnormal tracing; fibrin-based clot could not be reliably assessed
Day 1, early ROTEM tracing	INTEM	CT 34 s; A5 2 mm, A10 3 mm, A20 1 mm	Very low clot amplitude, suggesting profound clot firmness impairment
Day 1, early ROTEM tracing	HEPTEM	CT 427 s; A5 5 mm, A10 5 mm, A20 3 mm	Persistent low clot amplitude; severe hypocoagulability
Day 1, evening ROTEM tracing	EXTEM	CT 85 s	Relatively preserved extrinsic clot initiation
Day 1, evening ROTEM tracing	INTEM	CT >4875 s	Markedly prolonged intrinsic clotting time
Day 1, evening ROTEM tracing	HEPTEM	CT 18 s	Apparent correction; possible heparin effect, heparin contamination, or preanalytical interference
Day 1, evening ROTEM tracing	FIBTEM	CT 14 s; A5 15 mm, A10 17 mm, A20 15 mm	Fibrinogen-dependent clot firmness relatively preserved at this time
Day 2, late ROTEM tracing	EXTEM	CT 326 s; CFT 795 s; α 24°; A5 11 mm, A10 17 mm, A20 24 mm	Delayed clot initiation and impaired clot propagation
Day 2, late ROTEM tracing	INTEM	CT 379 s; CFT 869 s; α 22°; A5 10 mm, A10 16 mm, A20 23 mm	Global hypocoagulability with delayed clot propagation
Day 2, late ROTEM tracing	FIBTEM	CT 295 s; A5 3 mm, A10 4 mm, A20 4 mm	Markedly reduced fibrin-based clot firmness, consistent with impaired functional fibrinogen contribution
Day 2, late ROTEM tracing	HEPTEM	CT 582 s; CFT 1194 s; α 23°; A5 9 mm, A10 14 mm, A20 20 mm	Persistent coagulation impairment despite heparinase channel

Hemostatic management

Given the active bleeding phenotype and ROTEM findings, the patient received hemostatic resuscitation. Management included fibrinogen replacement, blood component therapy, tranexamic acid, and local hemostatic measures at bleeding puncture and catheter sites. Fibrinogen replacement was administered in the setting of active bleeding, hypofibrinogenemia, and markedly reduced late-phase FIBTEM amplitudes, indicating impaired fibrin-based clot firmness. Tranexamic acid was administered during uncontrolled bleeding. Blood components were transfused according to clinical evolution and laboratory abnormalities.

The rationale for ROTEM-guided therapy was that conventional coagulation tests confirmed severe coagulopathy but did not fully characterize clot quality, fibrin contribution, or whole-blood clot dynamics. ROTEM allowed bedside evaluation of clot initiation, propagation, and firmness, supporting a more targeted approach. In this patient, the late ROTEM profile was dominated by markedly impaired FIBTEM clot firmness, consistent with critical impairment of fibrinogen-dependent clot stability.

The main organ support and hemostatic interventions are summarized in Table 4.

**Table 4:** Hemostatic and organ support interventions Summary of the main therapeutic interventions provided during the patient's intensive care unit course. Management included airway protection, invasive mechanical ventilation, vasopressor therapy, corticosteroids, antimicrobial treatment, renal replacement therapy, metabolic support, and targeted hemostatic resuscitation. Fibrinogen replacement was guided by active bleeding, hypofibrinogenemia, and reduced FIBTEM clot firmness, consistent with fibrinogen-dependent clot failure in the context of hemorrhagic DIC. GCS: Glasgow Coma Scale; CRRT: continuous renal replacement therapy; KDIGO: Kidney Disease: Improving Global Outcomes; AKI: acute kidney injury; FIBTEM: fibrin-based thromboelastometry; DIC: disseminated intravascular coagulation

Intervention	Indication	Clinical rationale
Orotracheal intubation	GCS 6, hypoxemia, aspiration risk	Airway protection and oxygenation support
Invasive mechanical ventilation	Acute hypoxemic respiratory failure	Management of respiratory failure and aspiration-related pulmonary injury
Norepinephrine	Septic shock	Maintenance of perfusion pressure
Vasopressin	Refractory vasodilatory shock	Adjunct vasopressor support
Hydrocortisone	Refractory septic shock	Shock reversal strategy in vasopressor-dependent state
Broad-spectrum antibiotics	Suspected neuroinfection/pulmonary sepsis	Early infection control
CRRT	Anuric KDIGO 3 AKI, severe acidosis	Renal and acid-base support
Sodium bicarbonate	Profound acidemia	Temporary correction of severe metabolic acidosis
Dextrose infusion	Recurrent severe hypoglycemia	Glucose stabilization
Fibrinogen replacement	Low FIBTEM clot firmness, hypofibrinogenemia, active bleeding	Correction of fibrinogen-dependent clot failure
Tranexamic acid	Uncontrolled bleeding phenotype	Antifibrinolytic support when clinically indicated
Blood components	Active bleeding and DIC	Hemostatic resuscitation

Infectious management

The patient received antimicrobial therapy from the early course due to suspected severe infection with shock physiology. Initial antimicrobial coverage included ceftriaxone, later escalated to broader-spectrum therapy with a fourth-generation cephalosporin according to the clinical notes and evolving severity. The suspected infectious sources included central nervous system infection, aspiration-related pulmonary infection, and possible skin/soft tissue involvement.

The preliminary cerebrospinal fluid report of gram-positive cocci supported suspected neuroinfection, whereas the respiratory course supported aspiration-related pulmonary infection or pneumonitis. Blood cultures, respiratory sampling, and additional infectious workup were requested or pending during the clinical course. Despite antimicrobial therapy and intensive support, the patient progressed rapidly to refractory septic shock and multiorgan failure.

Clinical deterioration and terminal event

Despite maximal support, the patient's clinical trajectory remained unfavorable. He developed progressive refractory septic shock, severe tissue hypoperfusion, persistent hyperlactatemia, severe mixed acidosis, worsening hypoxemia under invasive mechanical ventilation, anuric renal failure requiring CRRT, overt hemorrhagic DIC, progressive cutaneous lesions compatible with purpura fulminans, and neurological deterioration with anisocoria.

During the final hours, the patient remained hemodynamically unstable despite high-dose vasopressor support. He showed poor peripheral perfusion, worsening neurological signs, ongoing bleeding, severe coagulation failure, and persistent metabolic instability. Family members were informed about the critical condition and poor prognosis. The patient subsequently developed witnessed asystole. Advanced cardiopulmonary resuscitation was initiated and continued for approximately 15 minutes without return of spontaneous circulation. An isoelectric tracing was documented, and death was declared at 10:37 p.m. on day 3.

A chronological summary of the patient's clinical course is presented in Table 5.

**Table 5:** Hemostatic and organ support interventions Summary of the main therapeutic interventions provided during the patient's intensive care unit course, including airway protection, invasive mechanical ventilation, vasopressor support, corticosteroids, antimicrobial therapy, renal replacement therapy, metabolic correction, and targeted hemostatic resuscitation. Fibrinogen replacement was guided by active bleeding, hypofibrinogenemia, and reduced FIBTEM clot firmness, consistent with fibrinogen-dependent clot failure in the setting of hemorrhagic DIC. GCS: Glasgow Coma Scale; CRRT: continuous renal replacement therapy; KDIGO: Kidney Disease: Improving Global Outcomes; AKI: acute kidney injury; FIBTEM: fibrin-based thromboelastometry; DIC: disseminated intravascular coagulation; CPR: cardiopulmonary resuscitation; ROSC: return of spontaneous circulation

Time point	Clinical event	Interpretation
Day 0/emergency department	Found unconscious after generalized tonic-clonic seizure; GCS 6; shock and hypoxemia	Severe acute neurological and systemic presentation
Day 0/emergency department	Orotracheal intubation; abundant gastrobiliary emesis during induction	Airway failure with high aspiration risk
Day 0	Cranial CT without acute structural lesion	Structural intracranial cause not identified
Day 0	Lumbar puncture performed; preliminary CSF report with gram-positive cocci	Suspected neuroinfection
Day 1/intensive care unit	Severe mixed acidosis, lactate elevation, AKI, thrombocytopenia	Early multiorgan dysfunction
Day 1	Cutaneous lesions documented over the dorsal-gluteal region	Initial skin injury, later suspicious for purpura fulminans
Days 1-2	Progressive shock requiring norepinephrine and vasopressin	Refractory septic shock
Day 2	Anuric KDIGO 3 AKI requiring CRRT	Severe renal failure in shock context
Day 2	Melena and bleeding from airway, oral cavity, puncture and catheter sites	Hemorrhagic DIC phenotype
Days 2-3	ROTEM deterioration with markedly low FIBTEM amplitudes	Fibrinogen-dependent clot failure
Day 3	Progression of purpuric/necrotic cutaneous lesions	Suspected purpura fulminans
Final hours	Persistent shock, neurological deterioration, severe hypoperfusion	Irreversible multiorgan failure
Terminal event	Witnessed asystole; CPR without ROSC	Death declared at 10:37 p.m. on day 3

Final clinical interpretation

This case represents a fulminant and catastrophic form of sepsis-associated coagulopathy progressing to overt hemorrhagic DIC, suspected purpura fulminans, refractory septic shock, and multiorgan failure. The initial presentation was dominated by acute neurological deterioration and generalized seizures, with suspected neuroinfection and possible toxicologic contribution from methamphetamine exposure. The respiratory course was complicated by aspiration risk and severe hypoxemic respiratory failure. The renal course progressed rapidly to anuric KDIGO stage 3 acute kidney injury requiring continuous renal replacement therapy, likely driven by shock, rhabdomyolysis, and systemic microvascular dysfunction [9]. 

The hematologic course was central to the case. Progressive thrombocytopenia, prolonged coagulation times, elevated fibrin-related markers, hypofibrinogenemia, gastrointestinal bleeding, airway bleeding, and bleeding from invasive sites were consistent with overt hemorrhagic DIC. The skin lesions evolved from erythematous-bullous lesions to violaceous, hemorrhagic, necrotic plaques compatible with purpura fulminans, reflecting severe dermal microvascular injury and systemic endothelial dysfunction.

ROTEM provided functional hemostatic information that complemented conventional coagulation tests. Serial viscoelastic assessment demonstrated progression toward advanced hypocoagulability, with late FIBTEM findings showing severely reduced fibrin-based clot firmness. This supported the interpretation of fibrinogen-dependent clot failure and guided fibrinogen replacement as part of hemostatic resuscitation. Despite invasive mechanical ventilation, dual vasopressor support, corticosteroids, antimicrobial therapy, bicarbonate therapy, continuous renal replacement therapy, blood component therapy, tranexamic acid, and fibrinogen replacement, the patient progressed to irreversible shock and cardiac arrest.

The case underscores the importance of recognizing septic DIC as a dynamic syndrome with thrombotic and hemorrhagic phenotypes, understanding purpura fulminans as a marker of severe endothelial and microvascular failure, and using ROTEM as an adjunctive bedside tool to identify functional coagulation deficits in critically ill septic patients with active bleeding.

## Discussion

This case illustrates a fulminant form of sepsis-associated coagulopathy progressing to overt hemorrhagic DIC, clinically suspected purpura fulminans, refractory septic shock, and multiorgan failure. The patient initially presented with acute neurological deterioration after generalized tonic-clonic seizure activity, severe hypoxemia, hypotension, tachycardia, metabolic acidosis, and early renal dysfunction. The initial diagnostic scenario was broad and included suspected central nervous system infection, aspiration-related pulmonary infection, sympathomimetic toxidrome related to methamphetamine exposure, rhabdomyolysis, acute kidney injury, and early sepsis-associated coagulopathy. However, the subsequent rapid progression to refractory shock, active bleeding, progressive thrombocytopenia, hypofibrinogenemia, purpuric-necrotic skin lesions, and ROTEM evidence of impaired clot formation supported septic DIC as the unifying pathophysiological process.

The most relevant feature of this case was the coexistence of thrombotic and hemorrhagic manifestations. The patient developed progressive cutaneous lesions clinically compatible with purpura fulminans, suggesting dermal microvascular thrombosis and hemorrhagic skin infarction. At the same time, he presented with active bleeding from the gastrointestinal tract, airway, oral cavity, catheter insertion sites, and puncture sites. This mixed phenotype reflects advanced septic DIC, in which systemic coagulation activation, endothelial injury, platelet consumption, coagulation factor depletion, fibrinolytic imbalance, and microvascular thrombosis may occur simultaneously. Therefore, the clinical course should not be interpreted as isolated bleeding or isolated skin injury, but as a systemic thromboinflammatory and consumptive coagulopathy associated with severe sepsis [3,11].

Sepsis-associated coagulopathy should be understood as a dynamic continuum. In early sepsis, coagulation activation may initially function as part of immunothrombosis, a host-defense mechanism involving thrombin generation, platelet activation, fibrin deposition, endothelial activation, leukocyte recruitment, and neutrophil extracellular traps. When this response becomes systemic and dysregulated, it promotes diffuse microvascular thrombosis, tissue hypoperfusion, organ dysfunction, and eventual consumption of platelets and coagulation factors. In this patient, the progression from shock physiology and early thrombocytopenia to overt bleeding, severe coagulopathy, suspected fibrinogen depletion, and multiorgan failure supports the concept that septic DIC is not a fixed laboratory diagnosis, but a time-dependent syndrome that may shift from thromboinflammation to hemorrhagic consumptive failure [2,11].

Overt DIC was supported by the combination of severe infection, shock, progressive thrombocytopenia, abnormal coagulation tests, elevated fibrin-related markers, hypofibrinogenemia, and clinically significant bleeding. The available clinical documentation supported an ISTH score compatible with overt DIC. Although different sections of the clinical record described variable score values, the overall clinical and laboratory phenotype was consistent with advanced consumptive coagulopathy. This distinction is important because DIC should not be diagnosed only by laboratory abnormalities; it requires interpretation within the clinical context. In this case, gastrointestinal bleeding, airway bleeding, oral bleeding, bleeding from catheter and puncture sites, progressive purpuric-necrotic lesions, and multiorgan failure strengthened the diagnosis of overt hemorrhagic DIC [2,12].

The serial cutaneous findings provided an important visual marker of disease progression. On day 1, Figure 2A showed multifocal dorsal and gluteal erythematous-violaceous lesions with epidermal detachment and hemorrhagic exposed dermis. On day 2, Figure 2B and Figure 2C showed enlargement of violaceous plaques, hemorrhagic bullae, necrotic-appearing areas, and extension toward the flank and gluteal region. By day 3, Figure 2D showed coalescent purpuric and necrotic lesions with extensive hemorrhagic skin involvement. This evolution was clinically compatible with a rapidly progressive microvascular thrombotic and hemorrhagic process in the context of septic DIC [3,4].

Purpura fulminans is pathophysiologically relevant because it represents the visible expression of systemic endothelial and microvascular failure. It is characterized by dermal vascular thrombosis, hemorrhagic infarction, and skin necrosis. In septic patients, this process may result from uncontrolled thrombin generation, endothelial activation, platelet consumption, impaired fibrinolysis, and loss of endogenous anticoagulant function. The initial differential diagnosis for the skin lesions included burn-like injury, pressure-related injury, immobilization-related soft tissue damage, and traumatic lesions related to the prehospital event. However, the rapid progression, violaceous discoloration, hemorrhagic bullae, necrotic features, and temporal association with overt DIC supported purpura fulminans as a major diagnostic consideration [3,4].

The protein C/protein S pathway is central to the biological interpretation of this phenotype. Under physiological conditions, activated protein C, with protein S as a cofactor, limits thrombin generation by inactivating factors Va and VIIIa and contributes to endothelial protection. During severe sepsis, endothelial injury, reduced thrombomodulin expression, inflammatory cytokine activity, natural anticoagulant consumption, and impaired endothelial protein C receptor signaling may contribute to acquired protein C pathway dysfunction. In this case, protein C and protein S levels were not available; therefore, acquired deficiency cannot be stated as laboratory-confirmed. Nevertheless, the combination of septic shock, purpura fulminans-like lesions, overt DIC, endothelial failure, and multiorgan dysfunction was biologically consistent with acquired impairment of endogenous anticoagulant pathways [3,4].

A key academic contribution of this case is the integration of serial ROTEM findings with the clinical phenotype of septic DIC. Conventional coagulation tests identified severe coagulopathy through thrombocytopenia, prolonged coagulation times, elevated fibrin-related markers, and hypofibrinogenemia. However, these tests provide limited information regarding whole-blood clot initiation, clot propagation, clot firmness, platelet-fibrin interaction, and fibrinogen-dependent clot strength. ROTEM provided functional bedside information that complemented conventional testing and allowed a more dynamic interpretation of the patient's hemostatic status [5,13].

The first ROTEM set obtained on day 1 showed extreme abnormalities, including absent or nonmeasurable parameters in EXTEM and FIBTEM and very low amplitudes in INTEM and HEPTEM, as shown in Figure 3A-3D. These findings suggested severe impairment of clot formation in the context of septic shock, acidosis, thrombocytopenia, evolving DIC, and active bleeding. Because of the extreme clotting times and nonmeasurable values, possible preanalytical factors, sampling issues, or sample interference should also be considered. This cautious interpretation is important because viscoelastic tests should always be correlated with the clinical bleeding phenotype, conventional laboratory tests, anticoagulant exposure, sample quality, and timing relative to transfusion or heparin exposure [5,14].

The second ROTEM set, obtained later on day 1, showed a different pattern, as shown in Figure 4A-4D. EXTEM clotting time was relatively preserved, whereas INTEM clotting time was markedly prolonged and appeared to correct in HEPTEM. This discordance raised the possibility of heparin effect, heparin contamination, or preanalytical interference. At that time, FIBTEM showed preserved fibrin-based clot firmness, with A5 15 mm, A10 17 mm, and A20 15 mm. This finding suggested that fibrinogen-dependent clot contribution was still relatively preserved during this intermediate phase, despite the severity of systemic illness [5,6].

By day 2, the ROTEM profile had deteriorated substantially, as shown in Figure 5A-5D. EXTEM and INTEM demonstrated prolonged clotting time and impaired clot propagation, while FIBTEM showed markedly reduced fibrin-based clot firmness, with A5 3 mm, A10 4 mm, and A20 4 mm. This late pattern was consistent with severely reduced fibrin-based clot firmness and impaired functional fibrinogen contribution in overt DIC with active bleeding. The temporal change from preserved FIBTEM clot firmness to markedly reduced FIBTEM amplitudes illustrates why serial viscoelastic testing may be more informative than a single isolated ROTEM assessment in rapidly evolving septic shock [5,6].

The late FIBTEM findings were clinically relevant because FIBTEM estimates the fibrin-based contribution to clot firmness after platelet contribution is inhibited. Very low FIBTEM amplitudes may reflect reduced functional fibrinogen contribution to clot firmness, impaired fibrin polymerization, or both. In the clinical context of active bleeding, thrombocytopenia, hypofibrinogenemia, DIC, and shock, this supported fibrinogen-dependent clot failure as a major contributor to the hemorrhagic phenotype. This also provided rationale for fibrinogen replacement as part of targeted hemostatic resuscitation [5,6].

Hemostatic management included fibrinogen replacement, blood component therapy, tranexamic acid, and local hemostatic measures. This approach was clinically justified by the presence of active bleeding, overt DIC, thrombocytopenia, suspected hypofibrinogenemia, and ROTEM evidence of impaired clot firmness. However, hemostatic therapy in septic DIC must be individualized. The goal is not to normalize every coagulation parameter, but to treat clinically relevant bleeding and correct the dominant functional defects contributing to hemorrhage. In this case, the dominant late abnormality was fibrinogen-dependent clot failure, rather than an isolated abnormality of platelet count or plasma coagulation time [5,14].

Tranexamic acid was used during uncontrolled bleeding and should be interpreted cautiously. Antifibrinolytic therapy may be considered in selected patients with clinically significant bleeding and suspected or documented hyperfibrinolysis, but septic DIC may also be associated with fibrinolytic shutdown, especially in thrombotic or early phases. In the absence of aprotinin-modified thromboelastometry (APTEM) or clear viscoelastic evidence of hyperfibrinolysis, tranexamic acid should be discussed as a rescue hemostatic intervention rather than routine therapy for septic DIC. This nuance is relevant because the most consistent ROTEM abnormality during the late phase was severe fibrinogen-dependent clot failure [2,5].

The patient developed a profound vicious cycle of shock, acidosis, hypothermia, and coagulopathy. Refractory septic shock caused tissue hypoperfusion, hyperlactatemia, endothelial injury, and microcirculatory failure. Severe acidosis may impair thrombin generation, fibrin polymerization, platelet function, and coagulation enzyme activity. Hypothermia can further impair platelet function and enzymatic coagulation reactions. Together, shock, acidosis, hypothermia, thrombocytopenia, and hypofibrinogenemia create a self-perpetuating cycle in which bleeding worsens oxygen delivery and hypoperfusion, while hypoperfusion worsens coagulopathy [2,15].

This explains why hemostatic therapy alone was unlikely to reverse the clinical trajectory. Fibrinogen replacement and blood component therapy can improve clot substrate availability, but their effectiveness may be limited when the patient remains profoundly acidotic, hypothermic, hypoperfused, and in refractory shock. Therefore, ROTEM-guided hemostatic resuscitation should not be separated from global septic shock management, including antimicrobial therapy, hemodynamic optimization, ventilation, temperature correction, metabolic support, renal replacement therapy when indicated, and ongoing reassessment of tissue perfusion [5,15].

The renal course was consistent with severe sepsis-associated acute kidney injury, progressing to anuric KDIGO stage 3 AKI requiring continuous renal replacement therapy. The mechanism was likely multifactorial, involving septic shock, hypoperfusion, rhabdomyolysis, possible methamphetamine-associated toxicity, convulsive activity, severe acidosis, and DIC-related microvascular dysfunction. Continuous renal replacement therapy was clinically appropriate because of anuria, severe acid-base disturbance, and progressive renal failure in a hemodynamically unstable patient. However, continuous renal replacement therapy in hemorrhagic DIC is particularly challenging because circuit anticoagulation may be unsafe in the presence of active bleeding and thrombocytopenia, while catheter placement and extracorporeal circulation may increase bleeding and thrombosis-related difficulties [9,16].

Methamphetamine exposure was clinically relevant in the initial differential diagnosis. Sympathomimetic toxicity can contribute to seizures, tachycardia, hypermetabolic stress, rhabdomyolysis, acute kidney injury, and metabolic acidosis. In this patient, methamphetamine use may have contributed to the initial neurological event, rhabdomyolysis, metabolic derangement, and renal injury. However, the later development of overt hemorrhagic DIC, purpura fulminans-like lesions, refractory septic shock, and progressive multiorgan failure was not explained by toxicologic exposure alone and supported severe sepsis-associated thromboinflammatory collapse as the dominant process [17,18].

The pulmonary axis was also clinically important because the patient presented with severe neurological impairment, seizure activity, abundant emesis, and subsequent hypoxemic respiratory failure. Aspiration pneumonitis or aspiration pneumonia may have contributed to the septic focus, particularly given the reported bilateral infiltrates and right basal consolidation. Although suspected neuroinfection was supported by the preliminary cerebrospinal fluid report of gram-positive cocci, the clinical course likely involved more than one inflammatory driver. Aspiration-related pulmonary injury, suspected systemic infection, skin barrier disruption, shock, and toxicologic-metabolic stress may all have amplified the inflammatory response [15].

Several limitations should be acknowledged. First, final microbiological confirmation of the infectious source was incomplete in the available documentation. The preliminary cerebrospinal fluid report supported suspected neuroinfection, but final culture details were not available. Second, protein C and protein S levels were not documented, so acquired dysfunction of this pathway can only be discussed as a biologically plausible mechanism rather than a laboratory-confirmed diagnosis. Third, some ROTEM tracings showed extreme values or discordant INTEM/HEPTEM behavior, so preanalytical factors, heparin contamination, sampling problems, or assay interference should be considered. Fourth, the available documents contained variability regarding the exact ISTH DIC score; therefore, the final reported score should be based on the definitive laboratory dataset selected for the manuscript [10,19].

Despite these limitations, the clinical phenotype remained highly coherent: severe sepsis, refractory shock, active bleeding, thrombocytopenia, hypofibrinogenemia, abnormal ROTEM findings, purpuric-necrotic skin lesions compatible with purpura fulminans, respiratory failure, renal failure requiring continuous renal replacement therapy, profound metabolic acidosis, and death from irreversible multiorgan failure. The case is academically relevant because it integrates multiple critical care domains and demonstrates that septic DIC may present with a mixed thrombotic and hemorrhagic phenotype. It also suggests that serial ROTEM assessment, particularly FIBTEM, may help characterize evolving impairment of fibrin-based clot firmness and support individualized hemostatic therapy in critically ill septic patients with active bleeding [6,12,14].

## Conclusions

This case demonstrates the fulminant progression of severe sepsis to refractory septic shock, overt hemorrhagic DIC, clinically suspected purpura fulminans, and multiorgan failure in a 30-year-old man. The coexistence of progressive purpuric-necrotic skin lesions, active hemorrhages, thrombocytopenia, hypofibrinogenemia, severe metabolic acidosis, acute renal injury requiring continuous renal replacement therapy, and respiratory failure reflected an advanced mixed thrombotic and hemorrhagic phenotype of septic DIC. Serial ROTEM assessment complemented conventional coagulation testing by demonstrating markedly reduced late FIBTEM amplitudes, consistent with impaired fibrin-based clot firmness and supporting fibrinogen replacement in the setting of active bleeding and hypofibrinogenemia. Despite aggressive organ support and hemostatic therapy, the patient progressed to irreversible shock and cardiac arrest, highlighting the poor prognosis of hemorrhagic septic DIC when associated with profound endothelial insufficiency, refractory hypoperfusion, and multiorgan dysfunction.
